# The impact of malocclusion on the prevalence of pain-related temporomandibular disorders in children and adolescents: a systematic review

**DOI:** 10.3389/fneur.2025.1550110

**Published:** 2025-04-03

**Authors:** Liliana Szyszka-Sommerfeld, Magdalena Sycińska-Dziarnowska, Karolina Gerreth, Gianrico Spagnuolo, Krzysztof Woźniak, Agata Czajka-Jakubowska

**Affiliations:** ^1^Laboratory for Propaedeutics of Orthodontics and Facial Congenital Defects, Chair of Maxillofacial Orthopaedics and Orthodontics, Pomeranian Medical University in Szczecin, Szczecin, Poland; ^2^Department of Maxillofacial Orthopaedics and Orthodontics, Chair of Maxillofacial Orthopaedics and Orthodontics, Pomeranian Medical University in Szczecin, Szczecin, Poland; ^3^Department of Risk Group Dentistry, Chair of Pediatric Dentistry, Poznan University of Medical Sciences, Poznan, Poland; ^4^Department of Neurosciences, Reproductive and Odontostomatological Sciences, University of Naples “Federico II”, Napoli, Italy; ^5^School of Dentistry, College of Dental Medicine, Kaohsiung Medical University, Kaohsiung, Taiwan; ^6^Department of Orthodontics and Temporomandibular Disorders, Poznan University of Medical Sciences, Poznan, Poland

**Keywords:** malocclusion, temporomandibular disorders, orofacial pain, pain-related temporomandibular disorders, TMD

## Abstract

**Background:**

The connection between occlusion and temporomandibular disorders (TMD) remains a topic of discussion, and current research does not provide a definitive answer. Pain-related TMD are the most common types of TMD. They include myalgia, arthralgia, and headaches attributed to TMD. This systematic review aims to synthesize the current scientific evidence regarding impact of malocclusion on the prevalence of pain-related TMD in individuals under 18 years.

**Methods:**

The study was based on a searching of the PubMed, Scopus, Embase, and Web of Science databases. Selection was limited to studies that explored the link between malocclusion and pain-related TMD in young individuals. The Newcastle-Ottawa Scale (NOS) was used to evaluate the quality of the selected research. The certainty of evidence was assessed using the Grading Recommendations Assessment, Development and Evaluation (GRADE) approach. A visual representation of the screening and inclusion processes was created using a Preferred Reporting Items for Systematic Reviews and Meta-Analyses (PRISMA) diagram. The information from the research was then summarized in a descriptive format.

**Results:**

Out of 868 potentially relevant articles initially identified, 13 studies were selected based on the inclusion criteria. The majority of these studies were determined to be of moderate quality, according to the NOS assessment. The certainty of evidence was assessed as low, according to the GRADE approach. Most of the studies showed that malocclusion may be associated with TMD-pain signs/symptoms in children and adolescents. The most frequent relationship was found between the prevalence of pain-related TMD and posterior crossbite, Class II malocclusion, Class III malocclusion, and anterior open bite.

**Conclusion:**

Certain types of malocclusion may have an impact on the prevalence of some TMD-pain signs and symptoms in young individuals. However, given the limited reliability of existing research and the inconsistencies observed across studies, additional well-structured, long-term investigations are necessary.

**Systematic review registration:**

https://www.crd.york.ac.uk/prospero/, identifier CRD42024570950.

## 1 Introduction

Temporomandibular disorders (TMDs) describe conditions affecting stomatognathic system structures, such as the temporomandibular joints (TMJ), masticatory muscles and other related structures. These conditions can present as tenderness or pain in the joints and/or muscles, sounds from the joints, and restricted or inconsistent jaw movement (1–3). Furthermore, TMDs can be accompanied by symptoms like headaches, neck discomfort, and dental pain. Pain-related temporomandibular disorders are the most prevalent forms of TMD. Discomfort can stem from the TMJ, the masticatory muscles, or adjacent structures. Typically, the pain occurs without prompting and intensifies with jaw motion and chewing (4–7).

The prevalence of TMDs, as well as their etiology, signs, and symptoms, varies according to age, race, and geographic location in different populations (8). Although TMDs are most prevalent in the 20–40-year age group, the presence of TMD signs and symptoms in individuals under 18 years of age ranges from 0.5 to 81% (8–12), with a female preponderance (8, 12–18). TMD symptoms tend to increase in prevalence and severity in particular in girls between the ages of 12–15 and this tendency continues with increasing age (1, 2, 19, 20). In young individuals, the two most frequently identified forms of TMD comprise muscle-related facial pain and disc displacement with reduction (12, 21–23). TMD-pain has been reported as the main non-dental cause of orofacial pain in subjects under 18 years of age. Pain-related TMD can impact a subject’s daily activities, psychosocial functioning and quality of life (24). Therefore, pain or dysfunction associated with TMD can negatively affect the well-being of children and adolescents. Pain-related TMDs can exist alongside other bodily pain issues, like headaches, neck aches, and backaches, and can also be linked to social difficulties, worry, low mood, and decreased academic achievement (25). It has been indicated that TMD may have an adverse impact on patients’ lives, manifesting as chronic pain, lack of energy, reduced physical activity, emotional disorders, poor sleep quality, changes in taste, discomfort when eating, and associated reduced life satisfaction (26, 27). Thus, as TMD can have serious consequences affecting a child’s everyday life, as well as their growth and development, early diagnosis in childhood can prevent more severe problems in adulthood. Additionally, as TMDs often go undetected in children, proper evaluation of these issues in this age group is crucial (28). The origins of TMD pain are complex, with various elements contributing to its development or persistence (21). These factors can encompass biological aspects like hormones, genetic predispositions, physical trauma, systemic disorders, occlusal interferences, parafunctional habits, and psychological influences like stress levels and emotional responses (29). Nowadays, mental health plays a dominant role in the etiology of TMD (30). An association has been found between painful TMD and moderate/severe levels of depression (31). It has been noted that hypertension and insulin resistance might influence the onset of TMD. Research indicates that elevated blood pressure can disrupt the central pain regulatory systems, potentially playing a role in pain-related TMD (32, 33). In addition, autoimmune and inflammatory disorders have been suggested to impact on the development of TMD. A connection was observed between the occurrence of TMD indicators and Hashimoto’s thyroiditis, hypermobile Ehlers–Danlos syndrome and hemophilia (32, 34–36). It has been reported a high occurrence of severe TMD in patients with synovitis, acne, pustulosis, hyperostosis, and osteitis (SAPHO) syndrome (37).

Alongside dental caries and periodontal diseases, malocclusion is one of the most common problems in dentistry (21, 38). The prevalence of malocclusion in people younger than 18 years old varies from 39 to 93% (8, 21, 28, 38, 39). The connection between TMD and occlusion is a common topic in research (38). Several authors have reported a relationship between different types of malocclusion and some TMD signs/symptoms. Malocclusion has been considered a risk factor for presence of clinical signs and self-reported TMD pain complaints in different populations (18, 40–44). Nonetheless, some research results have not provided clear answers on the topic (45). On the other hand, one study found no associations between malocclusion and pain-related TMD (46) and several other authors have questioned the possible influence of malocclusion on the development of TMDs (47–50). It should be emphasized that malocclusions addressed in different studies vary significantly. Furthermore, the correlations emphasized in certain research may only apply to specific forms of TMD, like facial muscle-related pain or disc displacement (18, 51). Consequently, the contribution of malocclusion to the onset of TMD remains a subject of debate.

Given the divergence in results between existing studies when it comes to the association between TMD and malocclusion, as well as high importance of TMD-pain problems in children and adolescents, a comprehensive overview of their findings is needed. As a consequence, this systematic review aims to summarize the current scientific evidence regarding the impact of malocclusion on the prevalence of pain-related TMD in children and adolescents. The hypothesis is that malocclusion may be a factor influencing the prevalence of pain-related TMD in individuals under 18 years.

## 2 Methods

To ensure clarity and compliance with established systematic review standards, the review plan was officially documented in the PROSPERO International Prospective Register of Systematic Reviews, bearing the registration number CRD42024570950. The review followed the recommendations outlined in the” Preferred Reporting Items for Systematic Reviews and Meta-Analyses” (PRISMA) guidelines (52).

### 2.1 Search strategy

A search of the four electronic databases (PubMed, Scopus, Embase, and Web of Science) was performed by two independent reviewers (L.S-S. and M.S.-D.), using the following keywords, first designed for PubMed: (“Pain”) AND (“Temporomandibular disorder*” OR “Temporomandibular joint” OR “Temporomandibular joint disorder*” OR “TMD”) AND (“Malocclusion”) AND (“Children” OR “Adolescents”). The strategy was modified for other databases, as is shown in Table 1.

**Table 1:** Search strategy.

Databases	Search strategy
PubMed	(“Pain”) AND (“Temporomandibular disorder*” OR “Temporomandibular joint” OR “Temporomandibular joint disorder*” OR “TMD”) AND (“Malocclusion”) AND (“Children” OR “Adolescents”)
Scopus	TITLE-ABS-KEY (“Pain”) AND TITLE-ABS-KEY (“Temporomandibular disorder*” OR “Temporomandibular joint” OR “Temporomandibular joint disorder*” OR “TMD”) AND TITLE-ABS-KEY (“Malocclusion”) AND TITLE-ABS-KEY (“Children” OR “Adolescents”)
Embase	(“Pain/exp”) AND (“Temporomandibular disorder*” OR “Temporomandibular joint” OR “Temporomandibular joint disorder*” OR “TMD/exp”) AND (“Malocclusion/exp”) AND (“Children” OR “Adolescents/exp”).
Web od Science	[All fields] (“Pain”) AND (“Temporomandibular disorder*” OR “Temporomandibular joint” OR “Temporomandibular joint disorder*” OR “TMD”) AND (“Malocclusion”) AND (“Children” OR “Adolescents”)

All relevant publications in the English language, regardless of their date of publication, was reviewed impartially. The final search, conducted on July 10, 2024, aimed to include all existing research. Additionally, references to the corresponding articles were collected manually to ensure a thorough search. Gray literature sources, including Open Gray, were also explored. The search was reiterated before the concluding analysis.

The PI(E)COS research questions for this systematic review were as follows: “Does malocclusion have an impact on the prevalence of pain-related TMDs in children and adolescents?” and “Is there an association between malocclusion and pain-related TMD in children and adolescents?”

Population (P): Children and adolescents irrespective of their gender and ethnicity.

Intervention/Exposure (I, E): Malocclusion irrespective of its type and severity and the methods used in its diagnosis.

Comparison/control group (C): Not applicable.

Outcomes (O): The presence of pain-related TMD; no restriction was applied in the study with regard to the type of symptoms and signs of TMD-pain, as well as with regard to the diagnostic criteria evaluating TMD signs and symptoms. Assessment of the impact of malocclusion on the prevalence of pain-related TMD. Analysis of the relationship between pain-related TMD and malocclusion in individuals under 18 years.

Study design (S): Observational studies.

### 2.2 Eligibility criteria

Inclusion criteria were as follows:

- Articles that cover the association between malocclusion and pain-related TMD in individuals under 18 years.
- Articles in which children and adolescents have no maxillofacial syndrome or a history of surgical interventions in the head and neck region.
- Observational studies that evaluate the relationship between malocclusion and pain-related TMD in children and adolescents.
- Full text articles are written and published in English regardless of the date of publication.

Exclusion criteria were as follows:

- Articles not covering associations between malocclusion and pain-related TMD in children and adolescents.
- Articles in which patients under 18 years of age have been diagnosed with maxillofacial syndrome and have undergone surgical interventions in the head and neck region.
- Articles examining the prevalence of pain-related TMD without any analysis of malocclusion in patients.
- Studies with ineligible designs, such as clinical trials, pilot studies, case reports, literature reviews, meta-analyses, research on animals, or unpublished data.
- Publications in languages other than English.

### 2.3 Extraction of data

After excluding duplicates and papers not written in English, the titles and abstracts of the remaining studies were initially reviewed by two independent reviewers (L.S.-S. and M.S.-D.) to pinpoint potentially eligible studies. Then, the full texts of the selected papers were thoroughly analyzed after taking into account the predefined inclusion and exclusion criteria. In each stage of the screening process the authors worked separately to ensure precision. Any disagreements during the review were settled by means of discussions held between the two authors and a third author (A.C.-J.), thereby ensuring a collective focus aimed at resolving any ambiguities and providing a thorough review process. Throughout this process, following information was extracted and collected: the relevant details of the author, the publication year, the country, the study design, the participants’ characteristics, including the mean age, gender ratio, total number of participants and the number of participants with TMDs, the criteria used for evaluating malocclusion and TMDs, and the main results. To allow easier comparative analysis of the included studies, the author conducting the final review (L.S.-S.) created spreadsheets based on the Cochrane Collaboration guidelines. When any information was lacking, the original study authors were contacted for supplementary details. If the requested data was not provided, the study was either removed or the missing information was labeled as “not reported” (NR). Cohen’s Kappa statistic was used to determine the degree of consensus among the reviewers.

### 2.4 Quality assessment

The Newcastle-Ottawa Scale (NOS) was used to evaluate the quality of the selected research (53). Since this systematic review only included cross-sectional studies, a modified NOS version suitable for cross-sectional studies was employed. This assessment covered three areas with seven criteria, utilizing a star-based system: selection (4 criteria, maximum 5 stars), comparability (1 criterion, maximum 1 star), and outcome (2 criteria, maximum 3 stars) (54). Scores of 0, 1 or 2 were awarded depending on whether the above criteria were not met, met or met using a validated method or an established model, respectively. According to the sum of these the overall study quality was deemed high (>7), moderate (5–7), or low (<5). The quality assessment process was carried out independently by two reviewers (L.S.-S. and M.S.-D.) after, engaging in discussions and consultations with a third author (A.C.-J.) to resolve any uncertainties or disagreements. The extent of consistency between the reviewers was determined through the calculation of Cohen’s Kappa value.

### 2.5 Data synthesis

A visual representation of the screening and selection process was created using a PRISMA diagram. Following a thorough review of the selected studies, critical data concerning study designs, participant demographics, interventions, outcome measurements, and key findings were collected. This facilitated a clear illustration of each study’s outcomes. After organizing the results into a table, a descriptive summary was developed. This summary considered the primary features of the study designs and participant groups, the frequency of malocclusion and TMDs, the nature of pain-related TMD symptoms, the type of malocclusion assessed (sagittal, vertical, or transversal), the method used to classify the type of malocclusion, the methods employed to evaluate the signs and symptoms of pain-related TMD, and a description of each study’s main conclusions.

Given the variations in how the original studies classified TMD pain, evaluated different types of malocclusion, or used different classification systems, and considering that some studies did not fully present their pain-related TMD data, conducting a meta-analysis was not feasible.

### 2.6 Certainty of evidence

The reliability of the evidence for each result was assessed using the Grading Recommendations Assessment, Development and Evaluation (GRADE) tool (55), which accounts for the types of studies included, potential biases, consistency of findings, relevance of evidence, accuracy of results, likelihood of publication bias, size of effect, dose–response relationship, and impact of potential remaining confounding factors. Since this study only presented a descriptive summary, the GRADE domains were rated according to the criteria outlined by Murad et al. (56) for systematic reviews without meta-analysis. Based on these ratings, the certainty of evidence was categorized as high, moderate, low, or very low.

## 3 Results

### 3.1 Study selection

The search strategy identified 868 potential articles: 277 from PubMed, 263 from Embase, 255 from Scopus and 73 from Web of Science. After eliminating 228 duplicate entries, 640 articles were retained. Following this, 609 articles were excluded due to their failure to meet the established criteria. Of these, 31 were eligible for a full-text evaluation. From the remaining 31, 18 were further excluded as they did not address the research question, leaving 13 articles for the final qualitative analysis.

**Figure 1:**
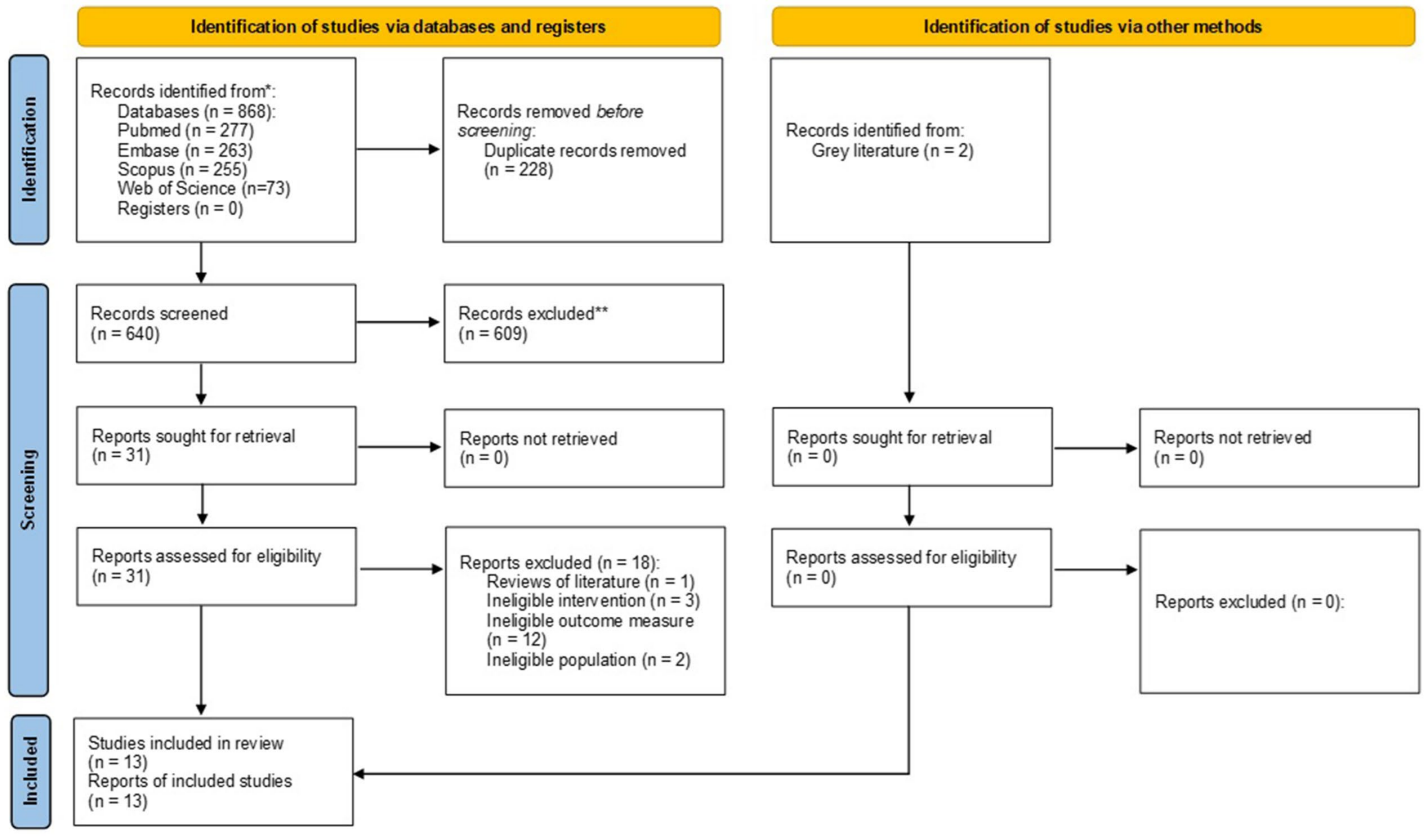
PRISMA flow diagram for the search strategy.

Figure 1, the Prisma Flow Diagram, visually depicts the entire search procedure, detailing each phase of the systematic review. The reviewers demonstrated a strong level of agreement, evidenced by a high Cohen’s Kappa coefficient of 0.96, indicating substantial consistency in their evaluations.

### 3.2 Characteristics of included studies

Table 2 compiles the principal characteristics regarding all the studies included in the review. All 13 of the studies were cross-sectional. Four studies were conducted in Brazil (51, 57–59), two in Turkey (40, 60), two in Columbia (18, 61), one in Italy (8), one in South Arabia (62), one in India (63), one in Croatia (64), and one in the US (65). The patients ages ranged in the age between 4 (59, 61, 62) and 17 (18, 65).

**Table 2:** Characteristics of the included studies.

Authors, Year (ref)	Study	Country	Total number of participants	Age of participants	% of females participants	% of participants with TMD	Type of malocclusion and the methods used in its diagnosis	Criteria for evaluating TMD signs and symptoms
Almaoudi, 2000 (62)	CS	Saudi Arabia	502	4–6 years	57.4%	16.9%	Clinical exam: sagittal discrepancies (anterioposterior relationship at the molar and canine level and overjet), transversal relationship (posterior crossbite), and vertical relationship (overbite)	Clinical exam: TMD sound, range of mandibular motion (MVO, opening deviation) and associated muscle disorders. TMD was diagnosed, if there is any single positive finding in the TMJ, the muscles and the opening criteria above.
Alpaydin et al., 2024 (60)	CS	Turkey	945	14.82 ± 2.06 years	66.0%	8.5% (2.9% with pain in TMJ area on palpation or mandibular movement or pain on palpation of the masticatory muscles, 3.7% with joint sounds, 1.4% with deflection, and 3.9% with deviation)	Clinical exam: sagittal malocclusion (Angle Class I, Class II, Class III) and transversal malocclusion (posterior and anterior crossbite)	Clinical exam: TMD was recorded based on the presence of at least one sign/symptom (sounds, pain, deflection/deviation, and limitation in mouth opening) as present or absent. TMJ sounds were evaluated by using a stethoscope. Pain in TMJ: pain in the TMJ area on palpation or mandibular movement. Pain in the muscles: pain on palpation of the masticatory muscles.
Bertoli et al., 2018 (51)	CS	Brazil	934	10–14 years	55.4%	33.08–43.68% depend on age; 8.2–18.6% with myofascial pain	Clinical exam: sagittal relationship, excessive overjet, anterior and posterior crossbite, open and deep bite	The presence of self-reported symptoms of TMD using a valid Portuguese version of the questionnaire by the AAOP. The clinical examination according to the RDC/TMD Axis I.
Bilgiç and Gelgör, 2017 (40)	CS	Turkey	923	7–12 years	50.2%	25.0% (1.8% with TMJ pain, 5.6% with clicking, 2.5% with masseter muscle pain, 4.2% with temporalis muscle pain)	Clinical exam: Angle Class, bimaxillary protrusion, overjet, overbite and posterior crossbite	Clinical records: the assessment of functional occlusion (anterior and lateral sliding, interferences), dental wear, mandibular mobility (maximal opening, deflection, deviation), and TMJ and muscular pain. The Clinical Dysfunction Index (Modified Helkimo Dysfunction Index) was used to measure the severity of TMD and the pain in the joints. TMD symptoms was recorded during interviews.
Caetano et al., 2024 (57)	CS	Brazil	580	7–8 years	49.1%	13.8% with TMD pain	DAI was used to categorize malocclusion (missing teeth in the anterior region, overjet, overbite, molar and canine occlusion)	Pain-related TMD was diagnosed using the 3-item version TMD pain screener included in the DC/TMD. The International Delphi Study – Part 1, reporting criteria for TMD pain using self-reported or proxy-reported TMD signs and symptoms in the last 30 days using questions related to the intensity of pain in the jaw or temporal area, the presence of pain or stiffness in the jaw on awakening and the presence of pain following activities in the jaw or temporal area.
Da silva et al., 2017 (58)	CS	Brazil	248	12 years	55.0%	26.6% (73% among participants with TMD had TMD-pain).	DAI was used to assess malocclusion	TMD symptoms were assessed using the questionnaire, recommended by the AAOP.
Mora-Zuluaga et al., 2022 (61)	CS	Columbia	77	4–14 years	67.3%	71.5% (33.8% with articular unilateral noise, 26.0% with pain of at least one masticatory muscle and 24.7% with pain in the TMJ)	Clinical exam: sagittal malocclusion (Class I, Class II/1, Class II/2, Class III), vertical malocclusion (normal, edge to edge, open bite, deep bite), and transverse malocclusion (posterior crossbite, scissor bite, normal), and presence of anterior crossbite	The HI was used with some modifications to determine the presence and severity of TMD.
Pereira et al. 2009 (59)	CS	Brazil	106	4–12 years	48.1%	12.26%	Clinical examination: Angle’s molar and/or canine classification (II or III) or relationship of primary secondary molars in distal or mesial relationship, incisor relationship (open bite, crossbite, overjet, or overbite), deviation from midline and posterior crossbite	Anamnestic questionnaire and clinical examination of signs/symptoms of TMD. The assessment of TMD was the presence of at least one sign or symptom, such as deviation during mouth opening, joint noises, movement limitation from pain or mechanical impairment, joint pain during movement.
Perrotta et al., 2018 (8)	CS	Italy	700	9–11 years	48.7%	14.7% with TMD-pain	Clinical exam: molar relationship, overjet, overbite and transversal molar discrepancy (unilateral or bilateral cross-bite)	The subjective evaluation of TMD-pain in the last 30 days by means of the Pain Screening Questionnaire to identify patients with orofacial pain using three questions related to the presence of pain, stiffness and pain during movement in the muscle and TMJ area.
Riolo et al., 1987 (65)	CS	US	1,342	6–17 years	50.3%	NR	Clinical exam: overjet, overbite, molar relation, and posterior crossbite.	Clinical examination and interview questions: Subjective symptoms on TMJ sounds, pain or discomfort in the jaws, headaches, earaches, and bruxism. Clinical signs of TMD: limited range of mandibular movement, mandibular locking or luxation, pain on movement of the mandible, joint sounds (clicking), and TMJ and muscle tenderness.
Sarit et al., 2019 (63)	CS	India	314	12–15 years	38.9%	28.7%	Malocclusion was assessed by means of the DAI	Signs and symptoms of TMD were analyzed by using a screening questionnaire recommended by the AAOP.
Šimunović et al., 2024 (64)	CS	Croatia	338	9–15 years	NR	42.0%	Clinical exam: the Angle classification system (normal occlusion and malocclusion Classes I, II, and III)	Clinical examination of TMD signs and symptoms (joint function and pain, masticatory muscles tenderness, range of mandibular motion, and joint sounds). Subjective evaluation on headaches, jaw locking, TMJ sounds, pain during mouth opening, or bruxism.
Thilander et al., 2002 (18)	CS	Columbia	4,724	5–17 years	49.8%	25.0% (tenderness to TMJ and muscles on palpation varied between 4 and 7%)	Clinical exam: Angle Class, bimaxillary protrusion, overjet, overbite and posterior crossbite	Clinical examination of TMD: the assessment of functional occlusion (anterior and lateral sliding, interferences), dental wear, mandibular mobility (maximal opening, deflection), and TMJ and muscular pain. A modified version of HI was calculated.

AAOP, American Academy of Orofacial Pain; CS, cross-sectional; DAI, Dental Aesthetic Index; DC/TMD, Diagnostic Criteria for TMD; MVO, maximum vertical opening; RDC/TMD, Research Diagnostic Criteria for TMD; HI, Helkimo Index; TMD, temporomandibular disorders; TMJ, temporomandibular joint.

The studies encompassed a total of 11,733 participants. The smallest sample was that included in the study of Mora-Zuluaga et al. (*n* = 77) (61), while the biggest sample appeared in the study conducted by Thilander et al. (*n* = 4,724) (18). When taking into account the gender of the participants, it is noticeable that girls were more commonly involved the subjects of research (51, 58, 60–62). Four studies featured similar percentages of boys and girls (18, 40, 59, 65). In three studies were the boys more numerous than the girls (8, 57, 63).

The overall prevalence of TMD symptoms/signs ranged from 8.5% (60) to 71.5% (61). TMD-muscle pain ranged from 2.5% (40) to 26.0% (61) and TMJ pain from 1.8% (40) to 24.7% (61). Two of the studies included patients with only TMD-pain diagnosis (8, 57).

The most frequently used method to diagnose TMD was a clinical assessment of TMD signs/symptoms (18, 40, 59–62, 64, 65). In three studies, the Clinical Dysfunction Index, a modified version of the Helkimo Dysfunction Index, was utilized to quantify the intensity of TMD and joint pain (18, 40, 61). One study employed the Research Diagnostic Criteria for TMD (RDC/TMD) in conjunction with a self-reported survey (51). Similarly, one study used the Diagnostic Criteria for TMD (DC/TMD) (57). The clinical protocols combined with a questionnaires were applied in five studies (18, 40, 59, 64, 65). Three of the 13 studies assessed the prevalence of TMD by means of a questionnaires (8, 58, 63) (two of them with a clinical examination of TMJ sounds) (58, 63).

In all the studies the presence of sagittal, vertical and/or transversal malocclusion was assessed thorough clinical examination (8, 18, 40, 51, 57–65). Three studies employed the Dental Aesthetic Index (DAI) to classify malocclusion based on severity and the necessity for treatment (57, 58, 63).

All of the studies examined the relationship between pain-related TMD and sagittal discrepancies (8, 18, 40, 51, 57–65). Eleven studies evaluated vertical discrepancies (8, 18, 40, 51, 57–59, 61–63, 65), and seven analyzed a transversal relationship (8, 18, 40, 51, 59–61).

### 3.3 Synthesis of results

Table 3 presents the key results from each study included in the review. A majority of the studies indicated a potential link between malocclusion and pain-related TMD in young individuals (8, 18, 40, 51, 59, 62–65). Six of these studies utilized a clinical evaluation of TMD signs and symptoms for diagnosis (18, 40, 59, 62, 64, 65). Bilgiç and Gelgör (40) diagnosed 1.8% subjects with TMJ pain, 2.5% with masseter muscle pain and 4.2% with temporal muscle pain. The authors observed strong associations between TMD signs and malocclusion, such as posterior crossbite, anterior open bite, Angle Class II and III malocclusions, and severe maxillary overjet. Similar results were observed by Thilander et al. (18). They found that TMJ and muscle tenderness on palpation varied between 4 and 7%. Alamoudi (62) found a correlation between the signs and symptoms of TMD (the authors assessed TMD sound, range of mandibular motion: maximum vertical opening, opening deviation and associated muscle disorders: TMJ and muscle tenderness) and occlusal characteristics, such as posterior crossbite, edge to edge, anterior open bite, and a Class III canine relationship. Pereira et al. (59) observed an increased risk for TMD signs and symptoms in posterior crossbite. TMD diagnosis was established if at least one sign/symptom was present, including deviation while opening the mouth, joint noises, restricted movement due to pain or mechanical issues, or joint pain during movement. Šimunović et al. (64) observed there is a significant impact of Class II malocclusion on the prevalence of TMD-pain. In this case, TMD signs were evaluated through a clinical examination, assessing joint function and pain, muscle tenderness, jaw movement range, and joint sounds. In turn, TMD symptoms were assessed through subjective reports of headaches, jaw locking, joint sounds, pain while opening the mouth, or bruxism. Similarly, Riolo et al. (65) assessed clinical signs of TMD, such as limited range of mandibular movement, mandibular locking or luxation, pain on movement of the mandible, joint sounds (clicking), TMJ and muscle tenderness and subjective symptoms on TMJ sounds, pain or discomfort in the jaws, headaches, earaches, and bruxism. The authors observed that open bite was linked to increased tenderness in the TMJ and surrounding muscles, while significant overjet, whether excessive or negative, was more likely to involve tenderness in the joint. Bertoli et al. (51) noted that 8.2–18.6% subjects had myofascial pain based on the Research Diagnostic Criteria for TMD (RDC/TMD) with a self-reported questionnaire. They found no association between TMD symptoms and an anteroposterior molar relationship or other types of malocclusion. However, in the case of myofascial pain, a significant and positive correlation was observed with an anteroposterior molar relationship. On the other hand, Perrotta et al. (8) and Sarit et al. (63) assessed the prevalence of TMD by means of a questionnaires (combined with a clinical examination of TMJ sounds) (63). The study of Perrotta et al. (8) focused on patients diagnosed with TMD-pain exclusively, utilizing the Pain Screening Questionnaire. This tool identified individuals with orofacial pain through three questions regarding pain, stiffness, and discomfort during movement in TMJ region and adjacent muscles. The authors found no association between pain-related TMD and sagittal discrepancies, e.g., molar relationship and overjet. However, these authors observed an increased risk of TMD in vertical and transverse discrepancies, such as negative overbite and unilateral and bilateral crossbite. Similarly, Sarit et al. (63) found that malocclusion assessed by means of the DAI was associated with TMDs.

**Table 3:** The results of the included studies.

Authors, Year (ref)	Results
Alamoudi, 2000 (62)	A significant correlation was found between signs and symptoms of TMD and occlusal characteristics, such as posterior crossbite (*p* < 0.05), edge to edge (*p* < 0.013), anterior open bite (*p* < 0.036), Class III canine relationship (*p* < 0.048), and asymmetrical canine relationship (*p* < 0.046).
Alpaydin et al., 2024 (60)	No significant differences were found between participants with and with no TMD for sagittal malocclusion (*p* = 0.120), anterior crossbite (*p* = 0.560), and posterior crossbite (*p* = 0.369).
Bertoli et al., 2018 (51)	There is no significant association between TMD symptoms and anteroposterior molar relationship or other malocclusions (*p* < 0.05). Adolescents Class II and Class III had higher prevalence of myofascial pain (95% CI 1.12–2.70; *p* = 0.015 and 95% CI 1.34–4.71; *p* = 0.004, respectively) than adolescents Class I.
Bilgiç and Gelgör, 2017 (40)	Moderate and severe dysfunctions were seen in individuals with Angle Class III malocclusion, an overjet greater than 0 and less than 6, and an increased overbite. Significant associations were found between different signs of TMD and posterior crossbite, anterior open bite, Angle Class II and III malocclusions, and extreme maxillary overjet.
Caetano et al., 2024 (57)	Malocclusion had no direct effect on either possible and probable sleep bruxism (*p* = 0.992 and 0.793, respectively) or complaints of TMD pain (*p* = 0.740). However, possible and probable sleep bruxism were associated with TMD pain complaints (*p* < 0.008 and *p* < 0.001, respectively).
Da Silva et al., 2017 (58)	No statistically significant association was observed between malocclusion and TMD symptoms (*p* = 0.4284).
Mora-Zuluaga et al., 2022 (61)	There was a statistically significant relationship between the severity of TMD and a transverse malocclusion (*p* = 0.016). No statistically significant differences were found in the rest of the variables. It was noted that individuals with Class I and Class II division 1 malocclusion reported more pain in at least one masticatory muscle and in the TMJ region compared to those with Class III malocclusion. Participants with an anterior crossbite experienced pain in the jaw joint and chewing muscles, along with deviation during mouth opening, predominantly characterized by a unilateral clicking sound in the joint. Those with a posterior crossbite exhibited a unilateral clicking sound and TMJ symptoms upon palpation.
Pereira et al., 2009 (59)	Univariate logistic regression analysis revealed that bruxism, posterior crossbite, midline deviation, finger sucking, Class II or III molar or canine malocclusion, and primary-molar distal step were significantly associated with the presence of TMD (*p* ≤ 0.30). However, only bruxism (OR = 6.08, 95% CI 1.51–24.5; *p* = 0.01) and posterior crossbite (OR = 5.74, 95% CI 1.18–27.85; *p* = 0.03) achieved statistical significance in the multivariate regression model and were therefore considered risk indicators for the presence of signs and symptoms of TMD.
Perrotta et al., 2009 (8)	A significant association was found between TMD-pain and a negative overbite (OR = 4.49, 95% CI 1.38–14.6; *p* = 0.020), unilateral crossbite (OR = 2.25, 95% CI 1.15–4.43; *p* = 0.006) and bilateral crossbite (OR = 2.47, 95% CI 1.10–55.53; *p* = 0.006). No significant association was found between TMD-pain and a molar relationship (*p* = 0.051) and between TMD-pain and overjet (*p* = 0.392).
Riolo et al., 1987 (65)	The results, as they pertained to occlusion and clinical signs, were as follows: functional shift was negatively associated with TMJ and muscle tenderness; open bite was positively associated with TMJ and muscle tenderness; excessive or negative overjet was more likely to involve joint tenderness. The results regarding occlusion and subjective symptoms were as follows: Class II molar relationship was positively associated with joint noise in 6–8 year olds and 15–17 year olds; subjects with a negative overjet were more likely to report joint noise.
Sarit et al., 2019 (63)	It was found that malocclusion was significantly associated with TMDs (OR = 0.71, 95% CI = 0.61–0.81; *p* < 0.001).
Šimunović et al., 2024 (64)	A binary logistic regression showed that only Class II malocclusion showed statistical significance impact on the TMD-pain. Class II malocclusion increased the likelihood by 2.6 times that the subject would exhibit at least one TMD symptom (OR = 2.61, 95% CI 1.16–5.83; *p* = 0.020).
Thilander et al., 2002 (18)	Significant associations were found between different signs of TMD and posterior crossbite, anterior open bite, Angle Class III malocclusion, and severe maxillary overjet.

TMD, temporomandibular disorders; OR, odds ratio; CI, confidence interval.

Three studies found no differences between TMD-pain and a specific type of malocclusion (57, 58, 60). More specifically, Alpaydin et al. (60) found no significant differences between participants with and without TMD when it came to sagittal malocclusion and anterior–posterior crossbite. These authors used the clinical protocol to assess TMD-pain. They reported that 2.9% participants had pain in TMJ region on palpation or during movement or pain on palpation of the masticatory muscles. In addition, da Silva et al. (58) and Caetano et al. (57) observed there is no impact of malocclusion assessed on the basis of the DAI on the prevalence of TMD-pain evaluated by means of the Diagnostic Criteria for TMD (DC/TMD) (57) or with using a questionnaires combined with a clinical examination of TMJ sounds (58). Caetano et al. (57) diagnosed pain-related TMD using a 3-question pain screening tool from the DC/TMD and the International Delphi Study – Part 1. This tool assessed TMD pain based on self-reported or proxy-reported signs and symptoms over the previous 30 days, focusing on the intensity of jaw or temple area pain, jaw pain or stiffness upon waking, and pain following jaw or temple area activities. Da Silva et al. (58) documented that 73% of participants with TMD symptoms, as evaluated by the Orofacial Pain and Temporomandibular Disorders Triage Questionnaire, a tool endorsed by the American Academy of Orofacial Pain (AAOP), experienced pain.

On the other hand, one study found a correlation between the severity of TMD and a transverse type of malocclusion, while no association was observed between any specific type of malocclusion and the occurrence of TMD (61). In the research conducted by Mora-Zuluaga et al. (61), 26.0% of the participants reported pain in at least one masticatory muscle, while 24.7% experienced pain in the TMJ. It was noted that individuals with Class I and Class II division 1 malocclusion reported more pain in at least one masticatory muscle and in the TMJ region compared to those with Class III malocclusion. Participants with an anterior crossbite experienced pain in the jaw joint and chewing muscles, along with deviation during mouth opening, predominantly characterized by a unilateral clicking sound in the joint. Those with a posterior crossbite exhibited a unilateral clicking sound and TMJ symptoms upon palpation.

### 3.4 Quality assessment

Table 4 consolidates the quality assessment results. A high level of consistency in the quality assessment was demonstrated by the two reviewers, as shown by a Cohen’s Kappa coefficient of 0.94. Using the NOS assessment for cross-sectional studies (54), four studies were evaluated as having a high quality (18, 40, 51, 57), while another nine studies were considered to have a moderate quality (8, 58–65). Despite this, differences remain in study designs, participant groups, and assessment techniques.

**Table 4:** The Quality Assessment of the studies using the adapted version of NOS for cross-sectional studies.

Authors, Year (ref)	Selection	Comparability	Outcome	Total score
Alamoudi, 2000 (62)	***	*	**	6
Alpaydin et al., 2024 (60)	***	*	**	6
Bertoli et al., 2018 (51)	****	*	***	8
Bilgic and Gelgör, 2017 (40)	****	*	***	8
Caetano et al., 2024 (57)	****	*	***	8
Da Silva et al., 2017 (58)	**	*	**	5
Mora-Zuluaga et al., 2022 (61)	**	*	**	5
Pereira et al., 2009 (59)	**	*	**	5
Perrotta et al., 2018 (8)	***	*	**	6
Riolo et al., 1987 (65)	**	*	**	5
Sarit et al., 2019 (63)	**	*	**	5
Šimunović et al., 2024 (64)	***	*	**	6
Thilander et al., 2002 (18)	****	*	***	8

### 3.5 Certainty of evidence

Table 5 details the reliability of the evidence regarding the connection between malocclusion and pain-related TMD, as assessed by GRADE. The overall quality of the studies’ evidence varied from “very low” (8, 18, 40, 58–65) to “low” (51, 57) for all measured outcomes.

**Table 5:** The results of the certainty of evidence for each outcome.

Outcome (method of TMD assessment)	Impact	Participants (studies)	Risk of bias	Inconsistency	Indirectness	Imprecision	Publication bias	Overall certainty of evidence	Comments
Clinical exam (with or without a question-naires) (18, 40, 59–62, 64, 65)	Significant impact reported in 6 studies	8,957 (8)	Serious	Not serious^b^	Not serious^c^	Not serious^d^	None^e^	⨁◯◯◯ Very low^*^	Malocclusion may have an impact on TMD-pain
RDC/TMD (51)	Significant impact reported in 1 study	934 (1)	Not serious^a^	Not serious^b^	Not serious^c^	Not serious^d^	None^e^	⨁⨁◯◯ Low^*^	Malocclusion may be associated with TMD-pain
DC/TMD (57)	No significant impact was reported	580 (1)	Not serious^a^	Not serious^b^	Not serious^c^	Not serious^d^	None^e^	⨁⨁◯◯ Low^*^	Malocclusion may not be associated with TMD-pain
Questionnaires (with or without a clinical assessment of TMJ) (8, 58, 63)	Significant impact reported in 2 studies	1,262 (3)	Serious	Not serious^b^	Not serious^c^	Not serious^d^	None^e^	⨁◯◯◯ Very low^*^	Malocclusion may have an impact on TMD-pain

DC/TMD, Diagnostic Criteria for TMD; RDC/TMD, Research Diagnostic Criteria for TMD; TMD, temporomandibular disorders; TMJ, temporomandibular joint; ^a^Most of the studies included presented a high quality; ^b^The studies included did not presented different directions of effect; ^c^The studies provided direct evidence to the research question; ^d^The optimal information size (≥400) was attended; ^e^None of characteristics, such as the body of evidence consisted of only small positive studies or when studies are reported in trial registries but not published were observed.

The magnitude of the effect, the plausible residual confounding factors and dose–response gradient were not rated; ^*^Certainty initially rated as “low” (a body of evidence consisting of observational studies).

## 4 Discussion

This systematic review of the literature presents the relevant findings regarding the impact of malocclusion on the prevalence of pain-related TMDs in young individuals. The review covered a total of 13 studies examining associations between TMD-pain and malocclusion in subjects under 18 years. Most of the studies were judged to have a moderate quality based on the NOS tool. The overall quality of the studies’ evidence was low, based on the GRADE criteria.

In general, studies show varied results, possibly due to differences in study designs, participant numbers, age ranges, cultural backgrounds, and evaluation techniques. Most research showed that certain types of malocclusion, such as sagittal, vertical and/or transversal malocclusion may be associated with pain-related TMD in children and adolescents (8, 18, 40, 51, 59, 62–65). However, their findings varied depending on the type of malocclusion studied, as well as on the types of TMD signs and symptoms occurring. The most frequent relationship was found between the prevalence of pain-related TMD and posterior crossbite (8, 18, 40, 59, 62), Class II malocclusion (40, 51, 59, 64, 65) and/or excessive overjet (18, 40, 65), Class III malocclusion or negative overjet (18, 40, 51, 59, 62, 65), and anterior open bite (8, 18, 40, 62, 65).

The findings of this review indicate that TMD varied in occurrence from 8.5% (60) to 71.5% (61), while TMD-related pain ranged from 1.8% (40) to 26.0% (61). These figures align with previous reviews, which reported an overall prevalence of TMDs in young individuals between 16 and 68% (10, 16). Similarly, the prevalence of pain-related TMD was consistent with other findings (11, 22, 66). However, the significant fluctuation in TMD prevalence could stem from variations in research methods, participant selection, and geographical location. It is also important to consider that TMD is frequently underdiagnosed in children. The lack of a standardized definition and the use of diverse diagnostic criteria across studies complicate the comparison of prevalence rates (16, 23, 67). In addition, in children under the age of 10 the prevalence of TMD is assessed on the basis of self-reported or proxy-reported signs and symptoms (3, 67). Furthermore, the way children describe their symptoms and react to physical examinations can present challenges, potentially affecting the accuracy of findings (68, 69). In this context, as early identification of TMD problems can prevent more severe problems during adolescence and in adulthood, an accurate diagnosis of TMD in children and adolescents based on a standardized and validated diagnostic instruments is especially important (23, 67). In this review, the most frequently used method to diagnose TMD was a clinical assessment of TMD signs/symptoms (18, 40, 59–62, 64, 65). The RDC/TMD were applied in one study (51), and one study used the DC/TMD (57).

Typically, TMDs were more common in female participants than in male participants (51, 58, 60–62). This observation aligns with other existing research (12, 15, 20, 70, 71). However, the connection between age and the occurrence of TMDs in young individuals remains unclear (72). There’s an indication that the likelihood of TMD in young people increases as they get older (2, 19, 23, 51) and that this may be a consequence of the increasing stress placed upon TMJ compared with its reduced adaptation mechanism (60).

The etiology of TMD is multifactorial. Bruxism was frequently cited as a contributing factor to TMD in growing individuals (21). Bruxism is defined as the activity of masticatory muscles during both sleep and wakefulness. Sleep bruxism involves rhythmic or non-rhythmic movements, while awake bruxism is characterized by repeated or sustained teeth contact and/or jaw clenching or thrusting. In healthy individuals, bruxism is not classified as a disorder, but rather as a behavior that can potentially lead to negative outcomes such as tooth wear, muscle pain, and damage of the oral mucosa (73, 74). While the connection between bruxism and TMD is intricate and not fully understood in young people and adults, it is widely believed that excessive bruxism can disrupt the masticatory system, leading to facial pain and TMD (21, 74–76). This is in line with our findings, where bruxism was associated with TMD-pain signs and symptoms (57, 59). In light of the above, it is essential that the diagnosis of bruxism must be accurate and based on reliable and validated screening tools. Research indicates that individuals with TMD tend to have more frequent premature tooth contacts and greater bilateral asymmetry in the occlusal force (77). Occlusal interferences are directly linked to pain in the masticatory muscles and instability in the TMJ, which can contribute to the development of TMDs. Premature contacts can cause condyle displacement, friction and increased intra-articular pressure on the TMJ, and as a consequence, the prevalence of TMJ and muscle disorders. Digital occlusal analysis in patients with TMD can help determine the connection between occlusal factors and TMJ problems, leading to more accurate diagnoses and better treatment planning (78, 79). As mentioned earlier, the link between occlusion and TMD is a recurring topic in research, but its influence on the development of TMD remains a subject of debate (48, 80). Previous reviews have concluded that a definitive connection between these two conditions cannot be established, and that other factors, such as injuries, non-functional habits, psychological factors, gender, genetic predispositions, and central pain mechanisms, are considered to be more influential (45, 81, 82). Nevertheless, the results of our study revealed a possible connection between posterior crossbites and the prevalence of some TMD signs and symptoms (TMJ pain, muscle pain and tenderness, TMJ sounds, clicking, and headache) in children and adolescents (8, 18, 40, 59, 62). This is consistent with previous findings. It has been observed that unilateral posterior crossbite and accompanying midline deviation are associated with signs and symptoms of TMDs (83). Also, Mora-Zuluaga et al. (61) identified unilateral posterior crossbite as the most common type of malocclusion associated with increased TMD severity. Notably, among various malocclusions, posterior crossbite is believed to significantly impact the proper function of the chewing system. This type of malocclusion disrupts the normal alignment of teeth and jaw muscles, potentially hindering chewing function by reducing the efficiency of food grinding (84, 85). It has been suggested that the altered occlusal relationship between the dental arches in posterior crossbite may result in asymmetric differences in the masticatory muscles and the condyle-fossa relationship, and as a consequence, this asymmetrical muscle activity could be the reason for their tenderness (45, 86). Additionally, changes in the alignment of the disc and condyle within the jaw joint caused by a posterior crossbite can lead to disc displacement and clicking sounds in the joint (87, 88). The potential link between posterior crossbites and TMD is particularly crucial for planning orthodontic treatment, as the occurrence of both conditions increases with age. Therefore, early orthodontic intervention for a functional unilateral posterior crossbite should prioritize correcting the irregular muscle activity and the altered position of the condyle caused by the misalignment of the mandible (83–85).

The studies also suggest a possible relation between Angle molar relationship and pain-related TMD. The authors observed that children and adolescents with Class II and Class III malocclusions had a higher prevalence of TMD-pain signs and symptoms (18, 40, 51, 59, 64, 65). In particular, a possible positive association was observed between Class III malocclusion or negative overjet and TMD (18, 40, 51, 59, 62, 65). These types of occlusal features may be a significant contributory factors in the deviation of TMJ components, as well as in masticatory muscle or joint tenderness (51, 65). Moreover, studies found that anterior crossbite and edge-to-edge anterior occlusion were associated with TMJ symptoms (62, 89). Hence, it is important that these types of occlusion in young individuals be treated orthodontically. On the other hand, whether, such orthodontic treatment will prevent or reduce TMD remains open to question. For these reasons, these patients should be the subject of longitudinal follow-ups to ensure adequate treatment planning in the future (18). Furthermore, the authors observed that Class II malocclusion may have an impact on the occurrence of pain-related TMD in children and adolescents (40, 51, 59, 64, 65). Research suggests that the lack of bilateral canine guidance during lateral excursions, especially in individuals with a Class II malocclusion, may increase the risk of developing TMD (64, 90). Previous review of the literature concluded that Class II malocclusion can be related only to muscle-related issues (81). This could potentially be attributed to changes in the functional occlusal relationship, leading to non-functional tooth contact, which is more commonly observed in individuals experiencing facial muscle pain (91). An additional factor could be the forward head position associated with Class II malocclusion. It is believed that this posture places additional strain on the muscles at the base of the skull and neck, potentially leading to muscle-related symptoms (92). Furthermore, recent research indicates that structural changes in the lateral pterygoid muscle, a muscle involved in jaw movement, observed in individuals with Class II malocclusion, could be connected to internal derangement and irregular disc movement (93). This can result in increased pressure within the joint, decreased lubrication, inflammation, and tissue damage, leading to pain, muscle soreness, and reduced height of the condyle and ramus, which are typical characteristics observed in individuals with Class II malocclusion (94). The results of our study also revealed a possible association between excessive overjet and the prevalence of some TMD signs and symptoms (TMJ and masticatory muscle pain and tenderness) in children and adolescents (18, 40, 65). This is in line with previous findings, where clinical signs of TMDs were significantly associated with increased overjet (95). In addition, it was found that excessive overjet that could be related with Class II malocclusion may stress the masticatory muscles and affect the displacement of the TMJ disk (42, 87, 96).

Similarly, the results of our study showed a correlation between vertical discrepancies, such as a negative overbite and anterior open bite and muscle and TMJ pain (8, 18, 40, 62, 65). Prior research has indicated that reduced overbite can lead to increased jaw movement, potentially overloading the joint and contributing to the development of TMD more often than in individuals with a greater overbite (97).

To summarize, it should be pointed out that some malocclusions seem to be associated with TMD-pain signs and symptoms in young individuals. During routine dental checkups, all children should receive a brief examination of the TMJ, masticatory muscles, and surrounding tissues. This particularly applies to individuals with Class II and Class III malocclusions, posterior crossbites, anterior open bites, and bruxism, as these factors seem to increase the risk of TMD-related signs and symptoms. While it is challenging to determine the likelihood of TMD based solely on the presence or malocclusion, early diagnosis and appropriate correction of malocclusion can leverage the effects of craniofacial growth to optimize functional adaptation. Hence, knowledge of the impact of malocclusion on the prevalence of TMDs in growing individuals could be beneficial for clinicians offering comprehensive treatment to children and adolescents as part of a preventative strategy for TMD. In light of the above, it is be recommended to pay special attention to interdisciplinary care of these patients, involving orthodontists, pediatric and general dentists working together in a coordinated manner. This multidisciplinary approach should utilize a variety of diagnostic and treatment methods. Embracing this concept of a multidisciplinary team is essential to improve the quality of life for these patients. However, it should be remembered that the association between occlusal factors and TMD is complex, involving a link of biomechanical, neuromuscular, and psychosocial factors. Therefore, it is essential to acknowledge the complexity of TMD etiology, which involves multifactorial influences beyond occlusal factors alone. All healthcare professionals should recognize the multifaceted and intricate nature of TMD and bruxism. They should be educated on the use of reliable and validated screening and assessment tools, and integrate these tools into their practice to provide appropriate care to patients before, during, and after any dental or orthodontic procedures.

This study aimed to better understand of the relationship between malocclusion and pain-related TMD in people younger than 18 years old. Given the high prevalence of malocclusion in this age group, as well as high importance of TMD-pain problems, such as their impact on daily activities, psychosocial functioning and quality of life in children and adolescents, our study provides a necessary and comprehensive overview of this issue. What is more, knowledge of the impact of malocclusion on the prevalence of TMDs in growing individuals may be important for clinicians in the interdisciplinary care of these patients. It is also important to note that this study assessed the certainty of the evidence for each outcome using the GRADE assessment tool to emphasizes the importance of considering the strengths and weaknesses of the evidence and the strength of recommendations in healthcare guidelines.

### 4.1 Limitations of the study

This systematic review has several limitations that need to be recognized.

(a) One limitation is that the overall quality of evidence in the studies reviewed was low, based on the GRADE criteria (the inclusion of non-randomized clinical trials introduces a potential bias, resulting in the inclusion of studies with a lower level of evidence and potential high bias); (b) It is also important to acknowledge certain limitations associated with using the Newcastle-Ottawa Scale (NOS) to evaluate the quality of the research. This tool does not take into account all possible source of biases or errors in study design and implementation; (c) Some of the analyzed studies were based only on a small group of children of varying ages (58, 59, 61); (d) Variations in the specific types of malocclusion examined and their degree of severity could potentially influence the research outcomes; (e) Variations in the methods used to assess TMD signs and symptoms, such as the use of questionnaires in some studies but not others, and inconsistencies in how clinical examinations were conducted, may have influenced the findings of some studies included in this review; (f) Most of the studies included both patients both with pain-related TMD and non-pain TMD diagnosis; (g) A significant limitation is the inclusion in the study both random samples and clinical orthodontic samples (children and adolescents from clinical orthodontic samples are more likely to have malocclusion compared to those from the general population); (h) An additional constraint is the cross-sectional design of all the studies, which only captures data at a specific moment in time. Consequently, considering these limitations, future research should involve long-term studies with larger participant groups, clearly defined diagnostic criteria, and rigorous scientific methods to enhance the understanding of this subject.

## 5 Conclusion

This systematic review provides a comprehensive overview of the impact of malocclusion on the prevalence of pain-related TMD in children and adolescents. Certain types of malocclusion, especially posterior crossbite, Class II malocclusion, Class III malocclusion and anterior open bite may be associated with the presence of TMD-pain signs and symptoms, such as TMJ and masticatory muscle pain and tenderness, and headache in subjects under 18 years of age. This information could be beneficial for healthcare providers offering comprehensive treatment to young individuals as part of a preventative strategy for TMD. However, considering the limited reliability of existing research and the differences observed across studies, additional long-term controlled investigations are necessary to validate the findings of this review.
